# 

**Published:** 1895-05

**Authors:** 


### NOTICES.

### Tri-State Meeting.

The Russell House, Detroit, which will be headquarters for the Tri-State Meeting, to be held June 18, 19 and 20, has made a rate of $2.50 and $3.00 per day according to location of room. The Hotel Normandie, an excellent house, just one block from the Russell, has made a rate of $2.00 and $2.50 according to room. Dr. W. C. Barrett, of Buffalo, will give a lantern lecture one evening during the meeting. Dr. Barrett has all of Prof. Miller's (of Berlin) bacteriological slides and those of Andrews on enamel formation. Dr. Hollingsworth, of Kansas City, will be present to demonstrate his system of crown and bridge-work. Railroad rates will be announced in the June numbers of the dental journals.

G. E. HUNT,
Secretary.

### Illinois State Dental Society.

Meeting to be held in Galesburg, May 14 to 17, 1895.

PROGRAMME—ESSAYISTS.

1. Address by the President, J. H. Cormany, subject: "The Saving of the First Tooth."
2. A. W. Harlan: "Dental Science and Literature."
3. J. Frank Mariner: "Dental Art and Invention."
4. Louis Ottofy: "A Review of the Transactions of the Illinois State Dental Society for a Quarter of a Century." Discussion opened by—.
5. C. B. Rohland: "A Simple Method of Keeping Records." Discussion opened by—.
6. D. M. Cattell: "Results of Experimental Root Canal Fillings." Discussion opened by Dr. E. K. Blair.

7. C. R. Taylor: "The Human Tongue." Discussion opened by Dr. Garrett Newkirk.

8. W. V. B. Ames: "Combinations of Metals for Amalgams." Discussion opened by Dr. E. D. Swain.

9. C. C. Southwell (Milwaukee, Wis.): "Compressed Air in Dentistry." Discussion opened by Dr. W. H. Taggart.

10. A. W. McCandless: "The Duties of Dentist to Patient. The Duties of Patient to Dentist." Discussion opened by Dr. A. H. McCandless.

11. E. H. Allen: "The Illinois State Dental Society and the Relation it Sustains to the Dentists of Illinois." Discussion opened by Dr. C. N. Johnson.

12. C. R. E. Koch: "A Commentary on the Illinois Dental Statute of 1881." Discussion opened by Dr. T. W. Brophy.

The following questions will be submitted to the society for discussion:

1. Can alveolo-dental abscess arise after complete sterilization and obliteration of the canal by an impervious filling, and if so, from what causes?

Dr. G. V. Black will open the discussion.

2. What are the best means of diagnosis of pulp calcification in its several forms; to what extent does the process demand treatment, and how shall it be treated (*a*) with respect to its prevention, (*b*) remedially?

Dr. E. Noyes will open the discussion.

3. What is the most satisfactory antiseptic, and the best method of root-canal sterilization?

Dr. J. W. Wassall will open the discussion.

4. Is not operative dentistry liable to the same injury from the too prevalent use of plastic stoppings as occurred to prosthetic practice from the introduction of vulcanite?

The discussion will be opened by Dr. W. A. Johnston.

Clinics will be given as follows:

1. L. E. Custer (Dayton, O.): "Electrical Fusing of Porcelain."

2. W. V. B. Ames: "Gold Inlay."

3. A. E. Matteson: "Porcelain Crown. Exhibit Furnace."

4. S. W. Lukin: "Bridge. Using Logan Crowns and Gold Crowns for Posterior Abutment."

5. T. W. Prichett: "Filling—Using Amalgam."

6. J. G. Reid: "Enlarging Root Canals with Sulphuric Acid."

7. I. A. Lumpkin: "Gold Filling in Disto-Proximal Compound Cavity of Molar or Bicuspid."

8. Josephine D. Pfeifer: "Gold Crown."

9. C. C. Corbett: "Regulating Teeth with the Jackson Crib."

10. H. Logan: "Cast Aluminum Plate."

11. L. W. Skidmore: "Gold and Tin Filling in Bicuspid."

12. A. W. Harlan: "Treatment of Pyorrhœa Alveolaris."

13. W. H. Taggart: "Porcelain Bridge. Exhibit Electric Furnace."

14. Grafton Monroe: "Management of Sodium and Potassium in Treating Putrid Pulp Canals."

15. G. E. Warren: "Gold and Platinum Filling in Bicuspid or Molar."

16. W. W. Tobey: "Immediate Separation and Filling of Incisor Teeth, Using William's Untrimmed Gold."

17. D. O. M. LeCron: "Porcelain Faced Crown, Giving in Detail the Method of Riveting the Same after the Gold Work is Finished."

18. G. H. Damron: "Gold Filling. Sibley's Mat Gold."

19. C. N. Thompson: "Will Make and Fit Downie Crown."

20. E. K. Blair: "Root Canal Filling."

21. F. H. McIntosh: "Will Make and Fit Crown, Using the Ludwig Anchor."

22. H. R. Staley: "The Use of Cements in Retaining Gold and Amalgam Fillings." Illustrated by models.

A. H. Peck, Chairman Ex've Com.
Louis Ottofy, Secretary.
Masonic Temple, Chicago.

### On to Baltimore—By What Way?

This question is in the minds of many, especially of those who are going to the meeting of the American Medical Association to be held in Baltimore on the 7th of May.

There are several routes, but none better, and we think none so good as the Chesapeake and Ohio Railway. This is one of the most picturesque and delightful routes in the country; the road throughout is in most perfect condition, it has no superior if an equal in this respect; its equipment is of the highest order. The attention and service, we have rarely seen equaled, one is made to feel at home from the moment he steps on the train. Of these things we speak from personal observation and experience. The general management of the road is such that one feels a sense of security not often experienced on some other roads. The scenery on this route is hardly equalled on any other road going eastward. The best train on which to make this trip is that which leaves Cincinnati at 12 o'clock each day and arrives in Baltimore at 8 o'clock on the next morning so that only an afternoon of daylight is occupied in making the journey.

All should bear in mind that this is the route for the meeting of the American Dental Association on the first Tuesday of August next, and don't forget it.

### CONTENTS.

COMMUNICATIONS.

The Origin of Pathological Tendencies.................................... 209

The Present Needs in Dentistry............................................... 214

MONTHLY DIGEST.

Operative Dentistry.............................................................. 237

SELECTIONS.

The Lay Press and the Medical Profession.................................. 236

Food for Thought.................................................................. 252

NOTICES.

Tri-State Meeting.................................................................. 253

Illinois State Dental Society.................................................... 253

The Horace Wells Permanent Memorial..................................... 256

Bibliographical..................................................................... 257

EDITORIAL.

Mississippi Valley Dental Association...................................... 257

Personal.—Mississippi Valley Dental Society............................. 259

On to Baltimore.—By What Way?............................................. 260

---

### THE DENTAL REGISTER,

A MONTHLY MAGAZINE DEVOTED TO THE INTERESTS OF THE

**DENTAL PROFESSION.**

*Terms Reduced to* $2.00 *Per Year. Single Copies,* 25 *Cents.*

**EDITED BY PROF. J. TAFT, D.D.S.**

—AND—

**Published by SAM'L A. CROCKER & CO., Ohio Dental and Surgical Depot.**

The volume commences in January and closes in December.

The REGISTER being issued monthly is always fresh, therefore Indispensable to the practitioner who desires to keep posted up with the new inventions and improvements which are daily developed. Neither expense nor effort will be spared to give value and variety to its contents. Articles will be illustrated with well executed engravings whenever they will add to the interest or assist to explanation.

Its extensive circulation and frequency of issue make it one of the BEST DENTAL ADVERTISING MEDIUMS in the United States.

All subscriptions must begin with January or July.

Local or special notices, five lines or less, will be inserted for $3 each insertion; over five lines, 50 cts. per line.

All advertising bills payable in advance, or at furthest, after the issue of the first number containing the advertisement. All transient advertisements to be paid for in advance.

☞CONTRIBUTIONS SOLICITED.

Exclusively Editorial Communications should be addressed to the Editor.

The latest number of the REGISTER may be ordered with or without other goods at any time, and all letters should be addressed to

